# Noncontact Heart and Respiratory Rate Monitoring of Preterm Infants Based on a Computer Vision System: Protocol for a Method Comparison Study

**DOI:** 10.2196/13400

**Published:** 2019-08-29

**Authors:** Kim Gibson, Ali Al-Naji, Julie-Anne Fleet, Mary Steen, Javaan Chahl, Jasmine Huynh, Scott Morris

**Affiliations:** 1 School of Nursing and Midwifery University of South Australia Adelaide Australia; 2 Electrical Engineering Technical College Middle Technical University Baghdad Iraq; 3 School of Engineering University of South Australia Adelaide Australia; 4 College of Medicine and Public Health Flinders University Adelaide Australia; 5 Neonatal Unit Flinders Medical Centre Adelaide Australia

**Keywords:** heart rate, respiratory rate, infant, electrocardiography, computers

## Abstract

**Background:**

Biomedical research in the application of noncontact methods to measure heart rate (HR) and respiratory rate (RR) in the neonatal population has produced mixed results. This paper describes and discusses a protocol for conducting a method comparison study, which aims to determine the accuracy of a proposed noncontact computer vision system to detect HR and RR relative to the HR and RR obtained by 3-lead electrocardiogram (ECG) in preterm infants in the neonatal unit.

**Objective:**

The aim of this preliminary study is to determine the accuracy of a proposed noncontact computer vision system to detect HR and RR relative to the HR and RR obtained by 3-lead ECG in preterm infants in the neonatal unit.

**Methods:**

A single-center cross-sectional study was planned to be conducted in the neonatal unit at Flinders Medical Centre, South Australia, in May 2018. A total of 10 neonates and their ECG monitors will be filmed concurrently for 10 min using digital cameras. Advanced image processing techniques are to be applied later to determine their physiological data at 3 intervals. These data will then be compared with the ECG readings at the same points in time.

**Results:**

Study enrolment began in May 2018. Results of this study were published in July 2019.

**Conclusions:**

The study will analyze the data obtained by the noncontact system in comparison to data obtained by ECG, identify factors that may influence data extraction and accuracy when filming infants, and provide recommendations for how this noncontact system may be implemented into clinical applications.

**International Registered Report Identifier (IRRID):**

RR1-10.2196/13400

## Introduction

### Background

The conventional method for monitoring vital signs of infants in the neonatal intensive care unit (NICU) is by the use of the electrocardiogram (ECG). This form of monitoring has disadvantages. One of the main concerns is that it is reliant on the use of electrodes that contain an adhesive layer [1]. The adhesive electrodes can cause damage to the fragile skin of preterm infants when removed and is the main source of skin breakdown in the NICU [2]. In addition, inaccurate apnea detection associated with artefact, generated by infant movement or cardiac activity, can influence monitoring accuracy [3].

Preservation of the integrity of the premature infant’s skin is essential and certainly a challenge in the NICU [4]. The immature layers of the skin of premature infants predispose them to skin trauma, specifically, owing to a reduced number of fibrils that are spread sparingly, inhibiting the connection of the dermis to the epidermis. Owing to the weaker dermal-epidermal junction, the skin can be injured more easily, particularly because of adhesive removal [5]. Adhesive dressings and tapes used in the NICU may form a stronger bond with the epidermis in comparison with the dermal-epidermal junction; therefore, when adhesives are removed, the entire epidermis can be stripped. In response to limitations, manufacturers have developed alternative products such as hydrogel electrodes that do not contain an adhesive layer; however, they may not secure to the skin as effectively and thus are potentially problematic in terms of monitoring reliability [6].

An alternative technique to the use of ECG is noncontact photoplethysmography (PPGi). The PPGi principle is based on the concept that light is absorbed by blood more than by the surrounding tissue. A pulse will therefore produce small variations in the color of the skin, indicating that blood is being circulated owing to the redness of hemoglobin. Researchers have been able to extract the heart rate (HR) by filming and magnifying this variation in skin color changes [7,8]. HR can also be detected from filming and magnifying the head motion of a subject. The internal carotid arteries supply oxygenated blood to the brain and the external carotid arteries supply the neck and face. Owing to pressure changes when blood is pumped through these major blood vessels, the head will oscillate by approximately 5 mm in amplitude [9].

To capture the respiratory rate (RR), movement of the chest wall can be observed. The chest rises from movement of the diaphragm during inspiration, typically from 4 mm to 12 mm [10]. To ensure the signals captured from skin, head, and chest variances after magnification are enhanced and clear of background noise, an improved video magnification technique can be applied. These algorithms have been previously described [11,12]. The end-product is a graphic panel of the 2 sources of data extraction using skin color and motion magnification (from the head or thorax).

In our previous study [13], the technique of motion magnification was tested using healthy children from the ages of 1 to 5 years who were situated in different lighting conditions with or without a blanket covering them. Noncontact data were compared with a respiratory belt transducer and commercial sleep monitor. This yielded positive results with a cross-correlation coefficient of .9812, making it suitable for biomedical applications. This technique needs to be further studied to determine feasibility with a larger sample size and by varying factors common to the neonatal population.

### Objective

The aim of this preliminary study is to determine the accuracy of a proposed noncontact computer vision system to detect HR and RR relative to the HR and RR obtained by 3-lead ECG in preterm infants in the neonatal unit. The specific objectives are as follows:

1. To determine if the noncontact system is accurate for measuring neonatal HR and RR when compared with ECG.
2. To identify factors that may influence data extraction and accuracy when filming preterm infants and provide recommendations for method improvement.
3. To provide an insight on how this noncontact system may be implemented for real-time and prototype development.

This technology may have the potential to replace conventional ECG and therefore avoid the adverse effects of its use with the preterm population.

## Methods

### Study Design

This is a single-center cross-sectional study of the proposed noncontact computer vision system based on quantitative methods. The concepts of PPGi and motion magnification will be applied to extract physiological data, with a statistical analysis of the results to quantify the relationship between the 2 methods of HR and RR monitoring using the Bland-Altman method [14].

### System Framework and Data Analysis

This section describes the framework used to extract HR and RR based on the physiological variations caused by cardiopulmonary activity from 2 different regions.

To extract the cardiac signal, it is necessary to amplify the subtle color variations on the skin surface using the standard Eulerian Video Magnification (EVM) method [15]. However, some modifications have been applied to the EVM method to suit the proposed monitoring system and provide some improvements related to reducing the execution time and motion artefacts.

These modifications include using a wavelet pyramid decomposition and an elliptic band-pass filter instead of the Laplacian pyramid decomposition. After amplifying the video, the region of interest (ROI) will be manually localized using the MATLAB built-in function *ginput*. The brightness values of the pixels within the selected ROI will then be spatially averaged to obtain pulsatile amplitude traces obtained from Red, Green, Blue (RGB) channels, as shown in the following equation:


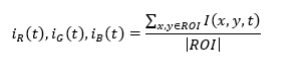


where *I (x,y,t)* is the brightness pixel value at image location *(x,y)* at time *(t)*, and |*ROI* | is the size of the selected ROI. As the G channel has the most apparent cardiac frequency band among other channels (R and B) [16-18], the *i*_*G*_ *(t)* signal will be selected to estimate the cardiac signal. A spectrum analysis approach based on the Fast Fourier Transform (FFT) will then be applied on the *i*_*G*_ *(t)* signal to transform it from the time domain to the frequency domain. The frequency band of interest (0.5-3 Hz) that corresponds to 30 to 180 bpm will then be selected using a separating ideal band-pass filter, followed by the inverse FFT to transform the filtered cardiac signal from the frequency domain to the time domain. Finally, peak detection based on the MATLAB built-in function, *findpeaks*, will be used to calculate the number of peaks of the acquired signal.

The variations in thoracoabdominal wall movement during respiration reflect directly in the spatial changes of intensity values in the video recording. The motion magnification approach proposed in previous work [11] will be first utilized to amplify the recorded video before data analysis. As the camera will capture the video in the RGB color space and to separate the intensity information from the color information, the RGB color space is converted to the YIQ color space using MATLAB’s built-in function called *rgb2ntsc*. The thoracoabdominal region will be manually localized where the cardiopulmonary signal is most pronounced using the MATLAB built-in function, *ginput*. The next processing step is to average the intensity pixel values over the frame sequences of the selected ROI from the Y channel of the YIQ color space, as shown in the following equation:


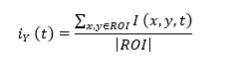


where *I (x,y,t)* is the intensity pixel value at image location *(x,y)* at time *(t)*, and |*ROI* | is the size of the selected ROI. A spectral analysis method using FFT is then applied to transform *i*_*G*_ *(t)* from the time domain to the frequency domain, followed by applying a separating ideal band-pass filter with selected frequencies of 0.15 to 2 Hz, corresponding to 9 to 120 breaths/min. The inverse FFT is then applied to transform the filtered breathing signal from the frequency domain to the time domain. Finally, peak detection based on the MATLAB built-in function, *findpeaks*, is carried out to identify the number of peaks of the acquired signal.

The measured value (*M*_*v*_) of the HR and RR per minute can be calculated using the following equations:


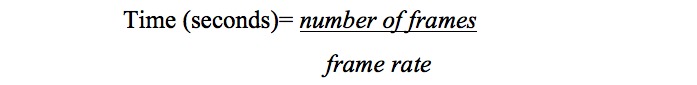


The period (p) between 2 peaks (average) is:


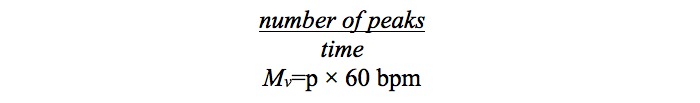


where p is the number of peaks of the acquired signal and t is the length of the video signal in seconds.

**Figure 1:**
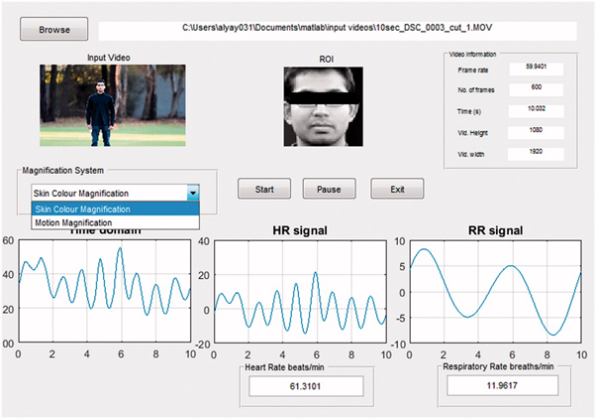
MATLAB graphic panel of the proposed system. HR: heart rate; RR: respiratory rate.

The end-product is a graphic panel of the 2 sources of data extraction using skin color and motion magnification. As shown in Figure 1, HR and RR are clearly presented in accordance to the selected timeframe.

### Participants

Participants will be selected using a convenience method. To assess the accuracy of the computer vision system in different situations, recruiting participants with confounding variables is necessary, such as respiratory support covering the face, variations in melanin concentration of infant skin, being cuddled by their parent, being positioned in an incubator, or receiving phototherapy producing a blue-light source. The inclusion criteria are as follows: infants below 37 weeks of corrected gestation who are monitored using the unit’s regular ECG monitor during the time of data collection to be used as a reference to validate accuracy of the proposed system. The exclusion criteria are as follows: term infants (≥37 weeks of gestation) or preterm infants that are not on ECG monitoring, who are likely to be discharged at the time of data collection, or who have major congenital abnormalities that could possibly make them identifiable in publications. Informed written consent will be obtained. Ethical approval for this study was obtained from the Southern Adelaide Local Network Research Committee (HREC/17/SAC/340; SSA/17/SAC/341) and the University of South Australia Human Research Ethics Committee (protocol number 0000034901).

### Sample Size

A sample size of 10 participants will provide 2 data points per participant (from PPGi and motion magnification), totaling 20 data points. This targeted sample size was determined given that this is a preliminary study investigating this novel technology still in its developmental phase. Results from this study will inform the rationale for conducting future large-scale studies.

**Figure 6:**
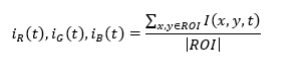
Calculation 1.

**Figure 7:**
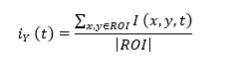
Calculation 2.

**Figure 8:**
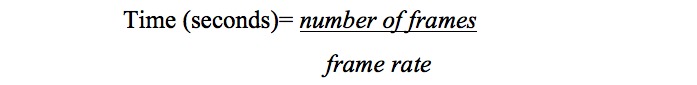
Equation 3.

**Figure 9:**
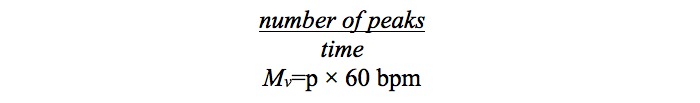
Equation 4.

### Data Collection

**Figure 2:**
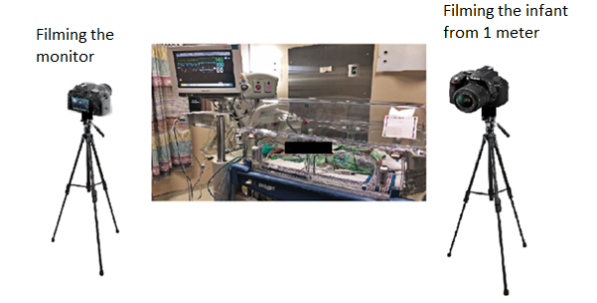
The experimental set-up for data collection.

Data collection will only commence after parents or guardians have acknowledged and signed the consent form provided. Participants will be filmed for approximately 10 min, which will provide adequate opportunity for data collection and variation in HR and RR. A Nikon D5300 and Nikon D610 camera will be used to film the infants and the cardiorespiratory monitor. Each camera will be mounted to a tripod and directed from approximately one meter away (Figure 2). The infant will not need to be repositioned for the purposes of the study as any sleeping position is acceptable. The researcher will ensure the ECG electrodes used for validation are correctly attached before filming. The entire body of the infant will be in the camera frame, with or without clothes or blankets, ensuring both methods of data extraction are possible. The infant will be filmed from various angles (eg, front on, from the back, and from the side) to assess accuracy from different perspectives.

Filming will take place by each camera concurrently, with the data and time of recording and participant number being noted. Clinicians will be able to have full access to the infant if required. If the infant was to have an apneic or bradycardic episode during filming, the clinician will be alerted by the unit’s ECG monitor. Filming will continue during this event unless directed otherwise by either the clinician or parent or guardian, because capturing significant variations in HR and RR will strengthen the efficacy of the noncontact system.

### Data Analysis

#### Phase 1

The first component of data analysis is data extraction using the noncontact system. The MATLAB environment—R2016a (MathWorks) with a Microsoft Windows 10 operating system will be utilized to conduct data analysis, producing the graphic panel for each participant (Figure 2).

Data points from both sources will be obtained at 3, 6, and 9 min for both RR and HR. A total of 3 data points will be determined as Altman and Bland [14] recommend that 2 or more measurements per participant be collected. The repeated measurements obtained from each participant will be averaged, which will not influence the bias between both measurements [19].

Owing to the potential for rapid fluctuations in infant HR and RR, the average HR and RR measurements over a 5-second period will be analyzed using the calculation previously described. The Phillips IntelliVue monitor that will be used to validate our results determines the HR by averaging the 12 most recent HR intervals from the ECG, and the RR is calculated by averaging the last 8 detected breaths [20].

#### Phase 2

**Figure 3:**
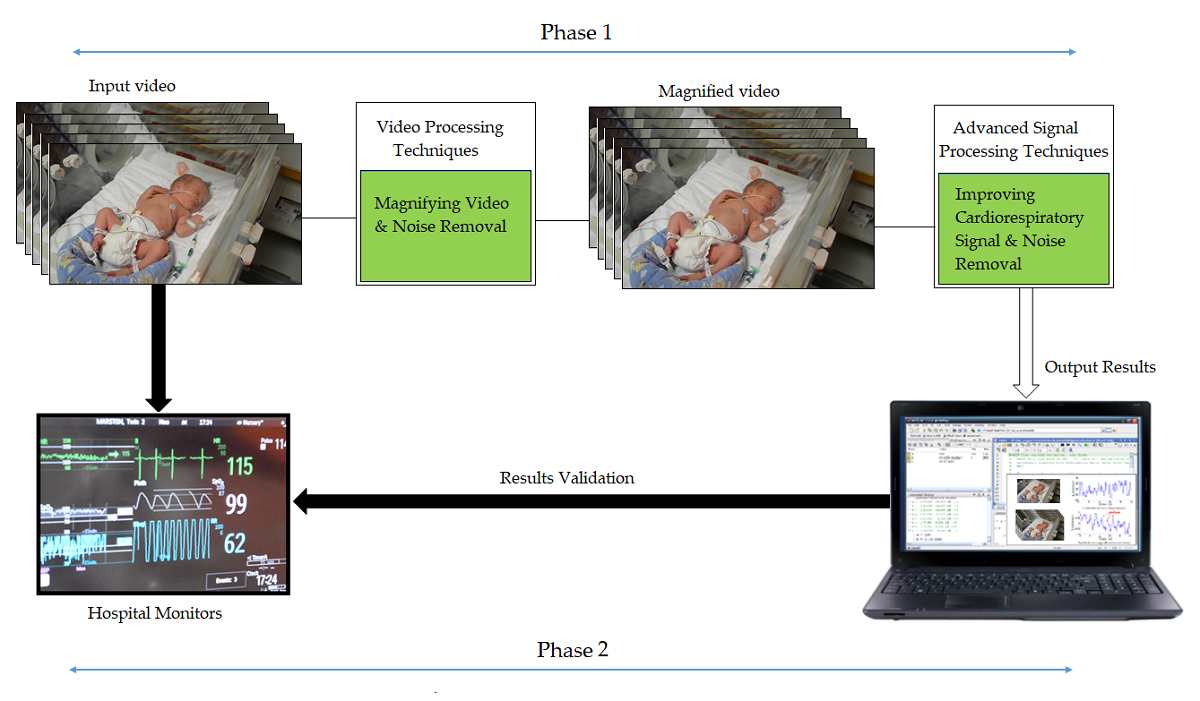
Data analysis flowchart.

The second component of data analysis is the statistical comparison of the 2 different methods. MedCalc Statistical Software version 18.2.1 [21] will be utilized to conduct this phase (Figure 3).

##### Clustered Regression Analysis

First, owing to collecting more than one measurement per participant, we cannot use a formal paired *t* test to test for zero bias between both methods. Therefore, clustered regression will be used to test the null hypothesis where the bias is zero.

##### Bland-Altman Analysis

Researcher KG will confirm the ECG readings at the selected intervals and compare the data extracted using the computer vision system which will be performed by researcher AA, limiting potential bias. A Bland-Altman analysis with multiple measurements per subject will be utilized from each method of data extraction (PPGi and motion magnification).

In total, 2 scatter plots (HR and RR) will be automatically formulated encompassing data for all participants using MedCalc software. The difference between the methods (input video vs ECG monitor; *i, d*_*i*_*=y*_*i2*_ *– y*_*i1*_) will be plotted against the average (*a*_*i*_*=(y*_*i1*_ *+ y*_*i2*_*)* /2) in the scatter plot [20 Stevens]. The formulation of this plot enables conclusions to be made with regard to whether the differences between the measurements are related to averages and to assess disagreement from possible error [14]. The scatter plot will be reviewed for possible errors such as proportional error. The mean bias between the two methods and the 95% limits of agreement as the mean difference (1.96 SD) are calculated [19]. Once determined, the mean bias will be compared with the clinically acceptable difference (CAD). The a priori criterion for bias and the CAD will be defined as an interval around zero (– *c,c*) [22]. Therefore the CAD should be close to zero for deeming the 2 methods as interchangeable.

## Results

Study enrolment and data collection began in May 2018. Results of this study have recently been published [23].

## Discussion

### Contribution to the Literature

We will be studying a real-life population in an environment that is characterized by variables demonstrated by other researchers to cause monitoring inaccuracy. Reduced environmental levels common in the NICU have been one of the main methodological challenges owing to impacting the PPGi signal to noise ratio with unwanted interference in related work [24]. Infants being covered by equipment or blankets have also been demonstrated to influence monitoring accuracy owing to difficulties in selecting an optimal ROI [1]. We have previously demonstrated accuracy with our algorithm to measure pediatric RRs in an environment with reduced lighting and with blanket coverings [13]. Therefore, our method may be able to address common accuracy issues with noncontact monitoring in the NICU.

### Limitations

A possible threat to our method’s internal validity is that the preexisting impedance monitoring will be utilized as the reference standard to validate data. Impedance monitoring can be influenced by patient movement or cardiac activity, thus impacting the RR signal [3]. Using the conventional ECG monitor to validate our results will be necessary to minimize disruption to participants for the sole purposes of this study. The application of additional modes for RR monitoring regarded as more accurate such as chest impedance belts or airflow detection methods will be considered for future large-scale studies [25].

The results will address the aim and objectives of this study and inform the design of future large-scale primary studies to refine methods, identify and address potential limitations of the technology, and evaluate the overall feasibility of the proposed noncontact system.

## Abbreviations

CAD: clinically acceptable difference
ECG: electrocardiogram
EVM: Eulerian Video Magnification
FFT: Fast Fourier Transform
HR: heart rate
NICU: neonatal intensive care unit
PPGi: photoplethysmography
RGB: Red, Green, Blue
ROI: region of interest
RR: respiratory rate
